# Editorial: Case reports in dermatology

**DOI:** 10.3389/fmed.2023.1269802

**Published:** 2023-08-23

**Authors:** Andreas Recke, Takashi Hashimoto

**Affiliations:** ^1^Department of Dermatology, Allergology and Venereology, University of Lübeck, Lübeck, Germany; ^2^Department of Dermatology, Osaka Metropolitan University Graduate School of Medicine, Osaka, Japan

**Keywords:** case report, autoimmune bullous diseases of the skin, rare diseases, allergy, drug reactions, autoinflammation, hereditary angioedema

## Introduction

Dermatology relies heavily on visual impressions, and dermatological residents need exposure to a variety of clinical pictures and presentations to develop their diagnostic skills. Moreover, the abstract immunological processes become more tangible and understandable when they are manifested on the skin. As such, case reports that feature unique or atypical visual characteristics are particularly valuable in Dermatology. Yet, it's crucial to emphasize that training budding dermatologists isn't solely about visual interpretations from case reports. The tactile nuances and three-dimensional aspects of skin conditions are just as vital, for some skin changes are better discerned through touch than sight alone. That said, our visual perception, augmented by tools like reflected-light microscopy, empowers us to discern intricate textures and color nuances, elements that two-dimensional images struggle to capture fully.

Case reports are typically considered the lowest level of clinical evidence (1). Although they require some clinical experience and specificity, they do not involve study design, experimentation, or targeted data collection. Because case reports are usually only published if they describe novel or unusual cases, they are often subject to bias. However, as a format, case reports do not limit themselves to a small number of variables, unlike randomized interventional trials, and can represent individual cases in their multifaceted nature.

To increase the transparency and quality of case reports, guidelines such as the CARE guideline have been developed (2). Some journals require adherence to these guidelines when submitting case reports, which can aid in the formal review process. However, Frontiers in Medicine does not mandate adherence to any specific guidelines for case reports.

While case reports do not have to be new or unusual to be published in Frontiers in Medicine, their scientific value may be reduced if there are studies with stronger evidence available. For example, if there are randomized controlled trials on the use of drug A for disease B, a case report on the same topic may not be necessary. The situation with the SARS-CoV2 pandemic has changed significantly over the past 3 years, with a wealth of new knowledge gained. As a result, case reports on SARS-CoV-2 that may have been interesting in the early days of the pandemic could now be obsolete or problematic. Ultimately, case reports should address gaps in medical knowledge, initiate discussions on diagnosis and treatment, and highlight areas where needs are unmet.

Frontiers does not impose a rate for rejections. Rejections are primarily justified on scientific grounds. In the case of this Research Topic, only 21 of 35 submitted case reports—and one correction—were finally accepted, which corresponds to a rejection rate of about 40%.

The articles in this Research Topic may be grouped into four thematic groups: autoimmune blistering diseases, cutaneous drug reactions and allergies, rare genetic diseases and miscellaneous reports.

## Autoimmune blistering dermatoses

This thematic group comprises seven articles that focus on cases of blistering dermatoses with unusual manifestations or associations with other diseases. Each article presents a unique case that sheds light on the clinical presentation, diagnosis, and treatment of these conditions.

For instance, Quattri et al. (a) [Corrigendum: Quattri et al. (b)], report on a 74-year-old woman with anti-laminin 332 mucous membrane pemphigoid (MMP) and severe pharyngolaryngeal involvement, who was successfully treated with topical and systemic therapy but later developed respiratory distress and required a tracheotomy.

Moro, Mariotti et al. describe a case of a 68-year-old woman with multiple sclerosis and scleroderma who developed bullous pemphigoid, and suggest that autoimmune diseases affecting the skin or organs where BP180 and BP230 are present could trigger the onset of bullous pemphigoid.

Moro, Ciccone et al. report on a second case: a 50-year-old man developed erosive lesions on the skin, oral mucosae and genital mucosae after Imiquimod treatment for superficial basal cell carcinoma. Diagnosis of pemphigus vulgaris was confirmed by immunofluorescence. Treatment with prednisolone led to complete remission in 4 weeks. Imiquimod therapy may induce pemphigus vulgaris in some patients. Sequential treatment should be considered for patients with multiple and large basal cell carcinoma to reduce the risk of adverse events.

In another article, Schauer et al. report on a male patient with a minimal manifestation of mucous membrane pemphigoid who presented with recurrent erosions in the urethral outlet area and gingiva, and who was diagnosed after a latency of around 4 years.

Kita et al. describe a case of a 15-year-old Japanese male who was initially suspected to have autoimmune bullous disease. However, the patient was ultimately diagnosed with prurigo pigmentosa and treated successfully with oral doxycycline hydrochloride hydrate and topical tacrolimus ointment. This case highlights that prurigo pigmentosa can mimick autoimmune blistering dermatoses including clinical, histological and immunologic aspects. This shows that the detection of autoantibodies does not necessarily imply their pathogenicity - and that all findings must be taken into account in order to come to a diagnosis.

The article by Didona et al. reports on a young patient with Behçet's disease who showed IgG autoantibodies against BP180, with the most common autoantigen in bullous pemphigoid, without developing blisters or urticarial-like plaques.

Finally, Minakawa et al. report on a case of a 20-year-old woman with autoimmune bullous disease (AIBD) who experienced mucocutaneous lesions after receiving the COVID-19 mRNA vaccine. The patient had IgG and IgM autoantibodies against epidermal basement membrane zone (BMZ) and a history of epidermolysis bullosa acquisita (EBA). Treatment with prednisolone resolved the lesions. The authors suggest that clinicians should be aware of the potential development of bullous pemphigoid-like AIBDs after COVID-19 mRNA vaccination.

## Cutaneous drug reactions and allergies

This topic covers 4 cases of toxic epidermal necrolysis and unusual forms of fixed drug eruption, as well as a case of LTP syndrome, which is relatively rare in Northern Europe in the described form.

The case reported by Paulmann et al. highlights the challenges of distinguishing two rare skin diseases, generalized bullous fixed drug eruption (GBFDE) and toxic epidermal necrolysis (TEN), and elucidates the importance of a distinct clinical presentation and detailed medication history. The patient, a 42-year-old male, presented with more than 50% skin detachment without defined areas of exanthema or erythema and a history of one prior event of TEN caused by acetaminophen, allopurinol or amoxicillin one and a half years before. The histology of a skin biopsy was unable to distinguish between the two diseases. The course of the disease, the later clinical presentation, and the medical and medication history, however, were in favor of a diagnosis of GBFDE. Metamizol was later on identified as the culprid drug because the patient developed a relapse after he received an additional dose of this medication.

Lin et al. describes a severe case of vanishing bile duct syndrome, a rare drug-induced disease characterized by cholestasis and ensuing ductopenia, in a patient who presented with concurrent Stevens-Johnson syndrome and hemophagocytic lymphohistiocytosis after the ingestion of non-steroidal anti-inflammatory drugs. Despite improvement in vanishing bile duct syndrome with steroid treatment, the patient died due to hypovolemic shock combined with septic shock episodes.

Zang et al. describe a rare case of TEN in a hepatitis A virus infection with acute-on-chronic liver failure patient. The patient's condition progressively worsened with a severe generalized rash, bullae and epidermal detachment accompanied by severe erosive mucosal lesions. The intravenous infusion of corticosteroids alleviated the patient's hypersensitivity, and the patient obtained lasting remission without severe adverse reactions and complications.

Otsuka et al. present the first case of a severe non-pigmenting fixed drug eruption (NPFDE) exhibiting general symptoms caused by chondroitin sulfate sodium. The patient of this case was a 44-year-old man who developed a severe rash and fever after taking chondroitin sulfate sodium. The rash was initially limited to the thighs but later spread to other parts of the body, and the patient developed systemic symptoms such as fever and arthralgia. The diagnosis of NPFDE was confirmed by skin biopsy showing infiltration of CD8-positive T-cells.

Albert et al. describe a rare case of a woman in Northern Europe who had an anaphylactic reaction to a meal containing various foods, including fruits, nuts, oats, wheat, and salmon. Allergy tests showed that the woman was not sensitized to Bet v1, which is common in birch food syndrome. Instead, she had a non-specific lipid transfer protein (nsLTP)-mediated food allergy, which is becoming increasingly common in Northern Europe. The authors suggest that LTPs should be considered as potential allergens, particularly for patients who experience severe reactions after consuming LTP-containing foods.

## Rare genetic diseases

Rare diseases clearly show the limits that are still faced by modern medicine (3). In the following articles, the authors describe how they faced this challenge of rare diseases and how they coped with it.

Costa et al. describe a case of a 30-year-old male patient, who underwent excision/grafting procedures for a giant congenital melanocytic nevus (CMN) at a child, and was diagnosed with metastatic melanoma more than 20 years later. The case highlights the importance of lifetime monitoring with once-yearly dermatological examination for large/giant CMN patients and the need for further clinical trials evaluating novel therapies for NRAS-mutant melanoma.

Hereditary angioedema with normal C1 inhibitor and unknown mutation (HAE-nC1INH-UNK), an exceedingly rare subtype of HAE, appears to be often misdiagnosed in patients who actually have mast cell-mediated angioedema. The article by Buttgereit et al. presents criteria for diagnosing HAE-nC1INH and emphasizes the importance of ruling out common differential diagnoses to reduce patients' disease burden and healthcare costs.

VEXAS syndrome is an autoinflammatory disease associated with severe inflammatory symptoms in adults. It is caused by somatic mutations in the UBA1 gene, leading to various clinical manifestations such as recurrent fever, skin conditions, vasculitis, and organ inflammation. A case study of a 64-year-old man with VEXAS syndrome presented by Tozaki et al. shows that treatment with oral prednisone and tocilizumab resulted in the resolution of symptoms. The study also measured derivatives of reactive oxygen metabolites (d-ROMs) as an indicator of oxidative stress and found that d-ROM levels decreased significantly after treatment.

Xue et al. report on a case of generalized pustular psoriasis (GPP) successfully treated with secukinumab, a monoclonal antibody that targets interleukin-17A. GPP is a rare and severe form of psoriasis that presents with erythematous, aseptic pustules, and common systemic symptoms include fever and myalgia.

## Miscellaneous reports

This thematic group comprises five unique cases describe unusual diseases, new diagnostic methods and new therapeutic options.

Scholl et al. describe a patient with long-term plaque psoriasis and psoriasis arthropathica who was treated with methotrexate and adalimumab. Then, this patient developed cutaneous pseudolymphoma caused by Leishmania infantum infection. Discontinuation of the anti-TNF-treatment resulted in resolution of the infection without anti-leishmanial therapy, highlighting the critical role of TNF in parasite control.

Yu Y. et al. describe a case of a 15-year-old boy with perifolliculitis capitis abscedens et suffodiens (PCAS), who was successfully treated with adalimumab and baricitinib, as the initial treatment of adalimumab and oral isotretinoin was insufficient. The proposed regimen is reported to be non-invasive and safe for treating PCAS.

Nie et al. report on a case of a 41-year-old Chinese woman who developed yellow-brown plaques in her eyebrows for several months following a tattoo in the same area. She was diagnosed with eyebrow tattoo-associated sarcoidosis, which was treated with topical corticosteroids with little effect. The treatment was a watch-and-wait strategy, including the recommendation to avoid permanent makeup.

Ha-Wissel et al. use optical coherence tomography (OCT) to monitor the individual treatment response of four patients with psoriasis or atopic dermatitis to biologic agents. Imaging parameters, such as epidermal thickness and vascular density, were used to enable objective quantification of inflammation in psoriasis or atopic dermatitis in selected representative skin areas. OCT potentially serves as an instrument to monitor biologic therapy in inflammatory skin diseases.

Yu Q. et al. applied chemical nail avulsion as a treatment for a 25-year-old male patient with onychomycosis. The conventional method proved to be effective in treating the patient, suggesting that chemical nail avulsion might be a viable alternative for the treatment of onychomycosis.

## Concluding remarks

The Research Topic “*Case Reports in Dermatology*” offers a collection of 21 insightful articles that shed light on the challenges of contemporary medicine. These case reports emphasize the importance of precise diagnosis and effective treatment, often demanding a multidisciplinary strategy. Beyond the insights gleaned from these textual and visual accounts, it's vital to recognize the inherent value of tactile and three-dimensional impressions in the educational journey of budding dermatologists. Engaging with real-world tactile experiences equips them with a comprehensive understanding, ensuring a holistic approach to dermatology. By assimilating knowledge from these cases and blending it with hands-on experiences, dermatologists can broaden their expertise, ultimately enhancing the quality of patient care.

## Author contributions

AR: Conceptualization, Writing—original draft, Writing—review and editing. TH: Conceptualization, Writing—original draft, Writing—review and editing.
